# Experimental results and mathematical formulation of non-spinning stone-skipping process

**DOI:** 10.1038/s41598-022-19992-x

**Published:** 2022-09-30

**Authors:** Hsuan-Wei Tsai, Hsieh-Chen Tsai, Wen-Fang Wu, Chun-Liang Lai

**Affiliations:** Department of Mechanical Engineering, National Taiwan University, Taipei, 10617 Taiwan, ROC

**Keywords:** Engineering, Mathematics and computing, Physics

## Abstract

Following a previously published paper in studying stone-skipping processes, detailed experimental figures are revealed in this paper. A mathematical model is also provided to explain the observed phenomena and measured data. The model separates the skipping process into several stages. It emphasizes, in particular, a hitting stage and a sliding stage, and also includes capillary-gravity wave resistance in its formulation. During these two stages, scale analysis is applied first to evaluate the relative importance among various forces acting on the stone. After reasonable simplification, a numerical algorithm is established to depict motion of the stone starting from its first hit of water to final sink. The total number of skips under specified initial throwing conditions can be predicted accordingly. The agreement between the analytical and experimental results indicates the applicability of the proposed model.

## Introduction

**Figure 1:**
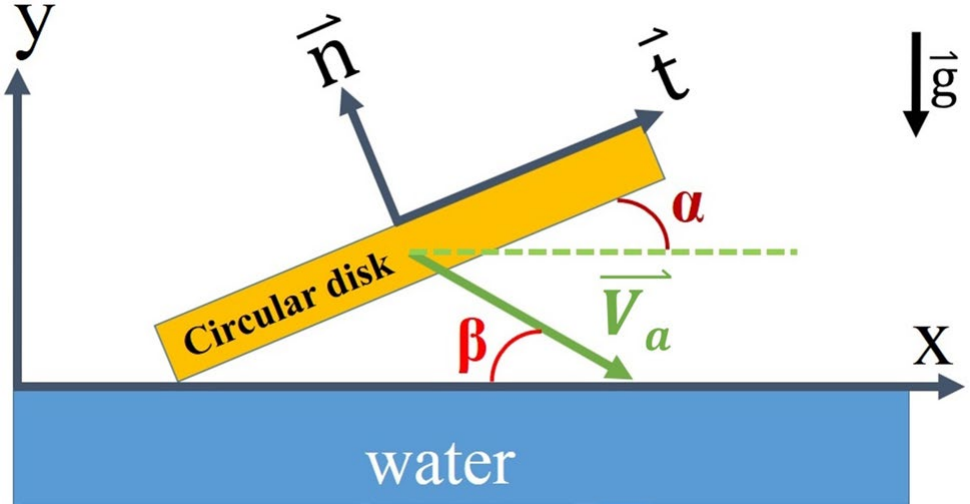
A circular disk resembling a stone approaching water surface.

Stone skipping has been a popular game since very old times. It is to throw a stone on a water pond to see how many bounces the stone can skip. Intuitively, people would pick a thin and flat stone of reasonable size to prevail in a contest. They would also throw the stone as fast as possible and with a small inclined angle. Intentionally or accidentally, people might throw the stone with a spin (rotation). Other than as a game, there have been many practical applications of stone-skipping in history, including the utilization in wars^1–3^. While many applications cannot avoid and therefore include the effect of spin, in other applications such as the drag boat racing, the seaplane landing and the emergency landing of airplanes on water surface, people try to avoid spinning of the boats and airplanes^4,5^. Therefore, it is important to study the stone-skipping problem, including the non-spinning stone-skipping process. In fact, the problem has been studied before by several researchers. However, most researchers considered the spinning effect and focused on theoretical developments and numerical solutions of the problem^6–8^. Only a few people paid their attentions to the study of non-spinning stone-skipping process^9,10^ and very limited experimental work can be found in the literature^11^. The major purpose of this paper is to release photos and measured data of our recent experimental work in studying the non-spinning stone-skipping process. To interpret the data, a new mathematical formulation and its numerical solution algorithm are also proposed. As pointed out by other researchers, the impact force is very crucial in studying the initial stage of entry of a solid into liquid^12–15^, it is specially considered in our formulation and numerical algorithm. For easy understanding of the afterward description and analysis, a schematic diagram is provided in Fig. 1 where $$\overset{\lower0.5em\hbox{$\smash{\scriptscriptstyle\rightharpoonup}$}}{{V_{{\text{a}}} }}$$ indicates the approaching or hitting velocity, $$\alpha$$ is called the inclined angle, $$\beta$$ is called the approaching or moving angle, and $$\mathop{t}\limits^{\rightharpoonup}$$ and $$\mathop{n}\limits^{\rightharpoonup}$$ are, respectively, the tangential and normal coordinates of a disk resembling a flat stone.

The basic physics and mechanism of stone skipping on water surface are not new to researchers. As stated in Bocquet^16^, the reaction force from the water surface when the stone hits it provides a lifting force on the stone to allow a rebound. In the meantime, the initial throwing velocity keeps the stone moving forward. By approximating the lift and friction coefficients to be one for most stone-skipping conditions, Bocquet described the motion of stone in *x*- and *y*-directions respectively as follows:

1 $$m_{s} \frac{{{\text{d}}V_{i,x} }}{{{\text{d}}t}} = - \frac{1}{2}\rho_{w} V_{i,a}^{2} A_{im} (C_{l} \sin \alpha_{i} + C_{f} \cos \alpha_{i} ),$$

2 $$m_{s} \frac{{{\text{d}}V_{i,y} }}{{{\text{d}}t}} = - m_{s} g + \frac{1}{2}\rho_{w} V_{i,a}^{2} A_{im} (C_{l} \cos \alpha_{i} - C_{f} \sin \alpha_{i} ).$$

**Figure 2:**
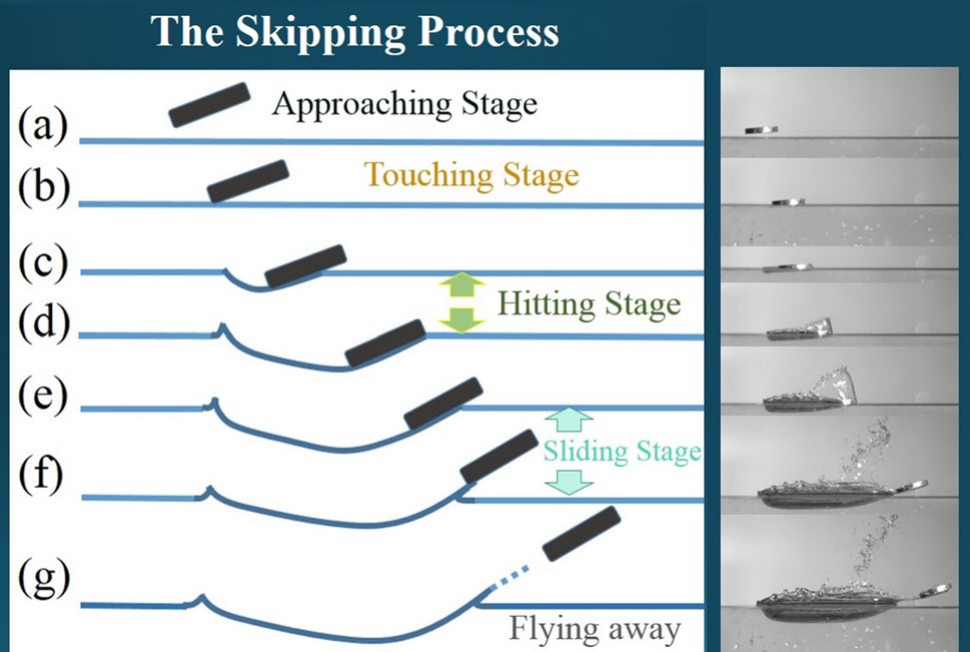
Detailed stages of stone-skipping: (**a**) approaching, (**b**) touching, (**c** & **d**) hitting stage, (**e** & **f**) sliding stage, and (**g**) flying away. (Initial throwing velocity $V_{0} = 5\;{\text{m}}/{\text{s}}$, inclined angle $\alpha = 4^\circ$ and approaching angle $\beta = 10^\circ$).

**Figure 3:**
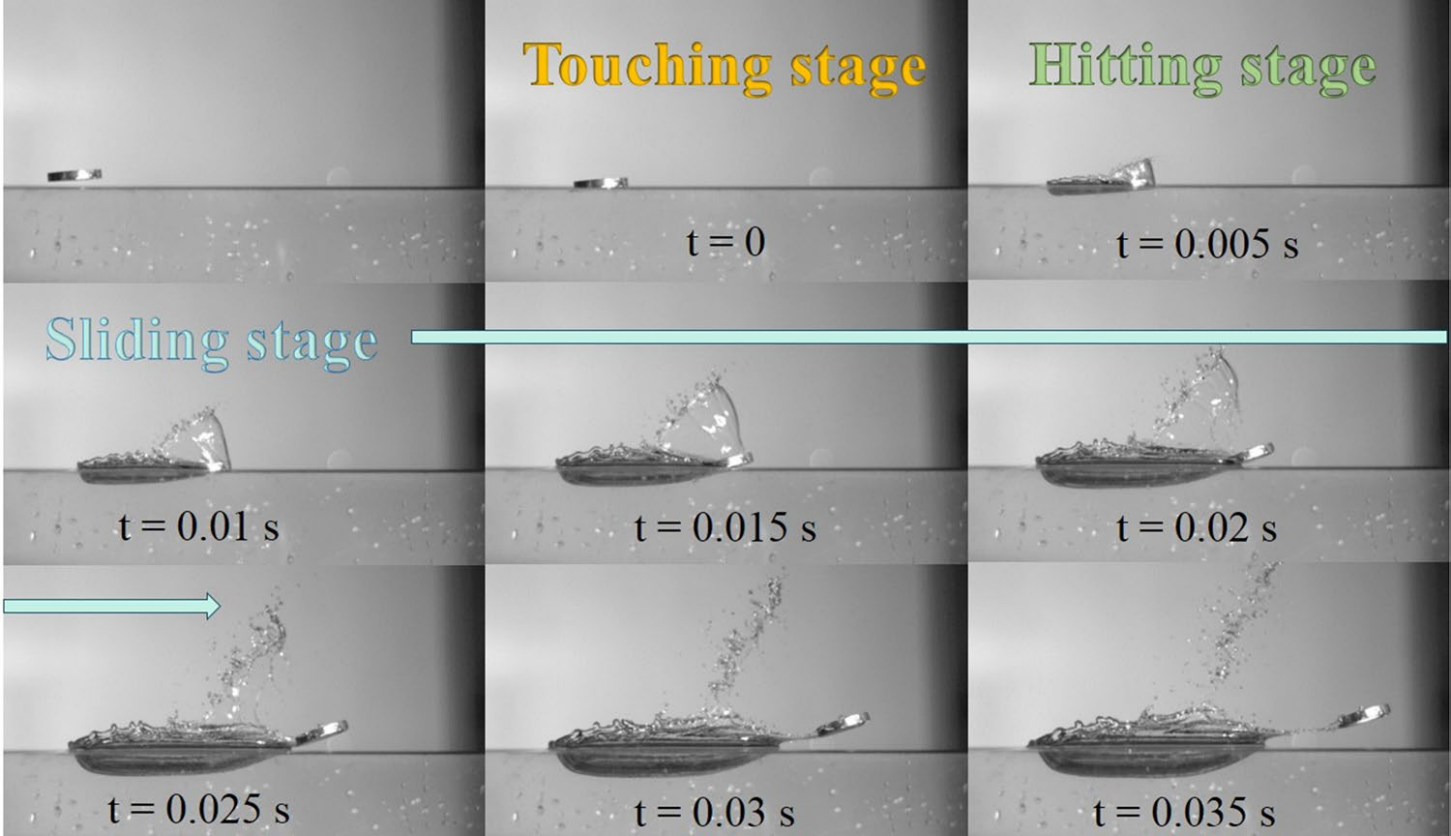
An experimentally observed stone-skipping process. (Initial throwing velocity $V_{0} = 5\;{\text{m}}/{\text{s}}$, inclined angle $\alpha = 4^\circ$ and approaching angle $\beta = 10^\circ$).

In the above equations, and also referring to Fig. 1, $m_{s}$ is the mass of stone, $\rho_{w}$ is the density of water, $g$ is the gravitational constant,$\alpha_{i}$ is the inclined angle of the *i*-th skip, $V_{i,a}$ is the magnitude of approaching velocity, $A_{im}$ is the immersed area of stone right after hitting, $C_{l}$ is the lift coefficient, $C_{f}$ is the friction coefficient, and the pair of ($V_{i,x} , V_{i,y}$) indicates the velocity of stone when it leaves water surface after the hit. Although the moving angle $\beta$ is shown in the figure, it does not appear in the equations. The angle will also be called approaching angle to describes the angle right before the stone hits the water surface. The above two equations of motion have been adopted by most of the people who intended to analyze theoretically the stone-skipping process. In fact, as that shown in Fig. 2 based on our experimental observation, it can be divided into 5 stages every time when the stone hits and then leaves the water during a stone-skipping process. Our experimental setup contains a throwing machine, four separated water tanks aligned at suitable distances away from the throwing machine, a high-speed camera (Olympus ix Cameras, 2000fps in 1024 × 768 resolution with lens of Nikon 24–85 mm and led of 10 W), and a computer. An aluminum circular disk having 3 cm in diameter, 5 mm of thickness and 9.0 g of mass was thrown from the throwing machine toward the water surface of the first tank to generate the stone-skipping process, and the process repeats many times. The skipping processes of the circular disk on the water surface were recorded by the high-speed camera. By observing closely our experimental results in Figs. 2 and 3, we found the hitting stage and sliding stage are the most two important stages when the stone interacts with the water. However, the physical mechanisms and forces acting on the stone at these two stages are not quite the same. Describing the above motion by only Eqs. (1) and (2) may not be appropriate. Other factors have to be taken into consideration, and it is also the major drive for writing this paper.

**Figure 4:**
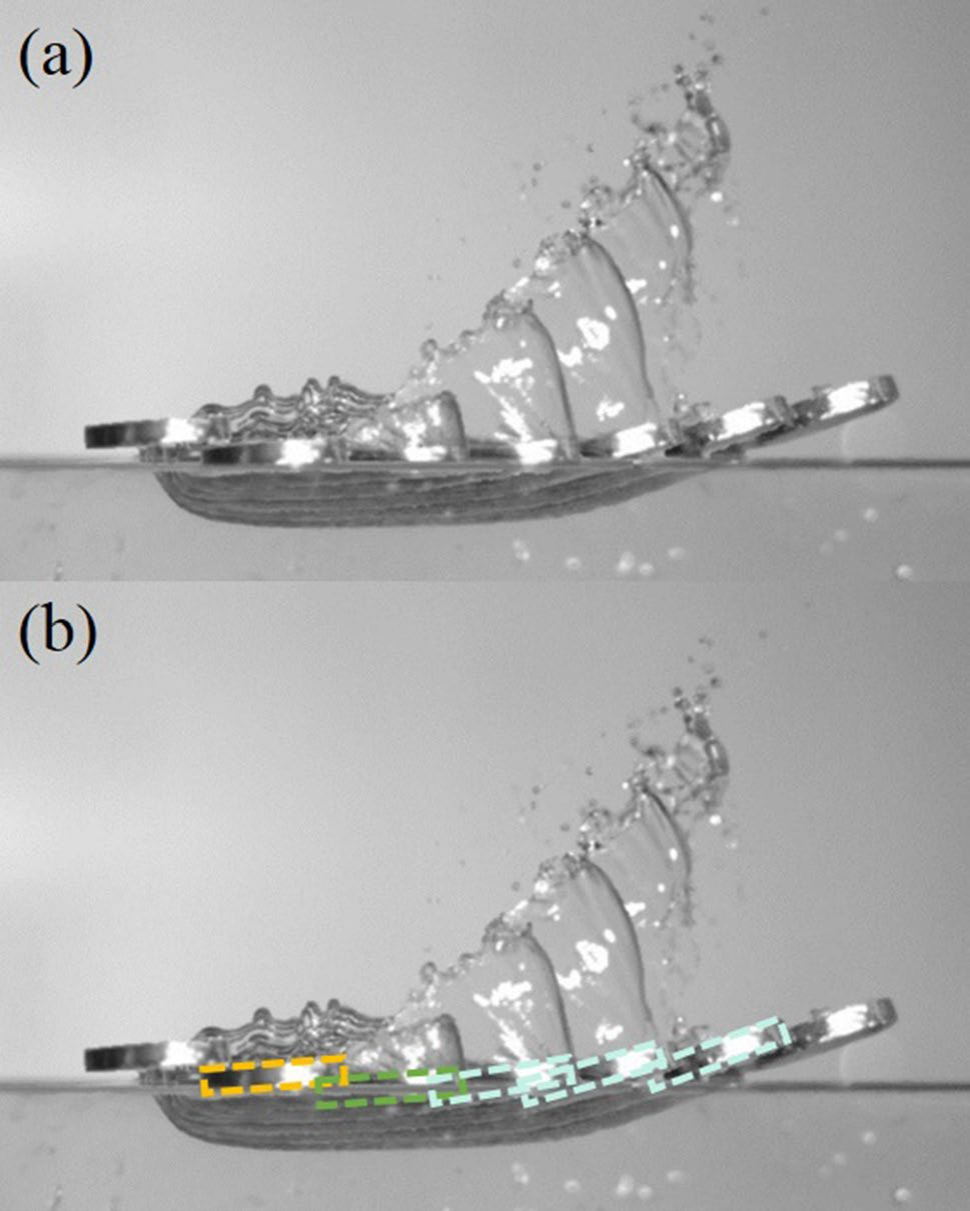
An overlapping image of stone-skipping: (**a**) the original image; (**b**) division of approaching, touching (in orange), hitting (in green), sliding (in light blue), and flying-away stages.

With a close observation of Figs. 2 and 3, and the overlapping image of Fig. 4, the stone keeps descending after it hits the water surface in the hitting stage and, in the meantime, moves and pushes the water forward. In this stage, the forces acting on the stone include the weight of stone, the lifting force from the reaction of water surface, and the capillary-gravity wave drag. In the sliding stage, the stone starts to ascend but also slides along and finally skips off the water surface. In addition to the gravitational force and the capillary-gravity wave drag, there exists frictional resistance (also called “skin friction”) when the stone slides along the water surface. Since the stone starts to ascend and move away from the water in this stage, the reaction force from the water surface does not exist. Nevertheless, an impulse owing to the reaction of water surface during a very short-time transition interval still provides the stone an upward velocity. It is felt the mathematical model of stone-skipping process can be reformulated by considering separately the above two stages.

## Modelling

Based on description of the previous section, a mathematical model different from Eqs. (1) and (2) of Bocquet’s is described as below.

### The hitting stage

**Figure 5:**
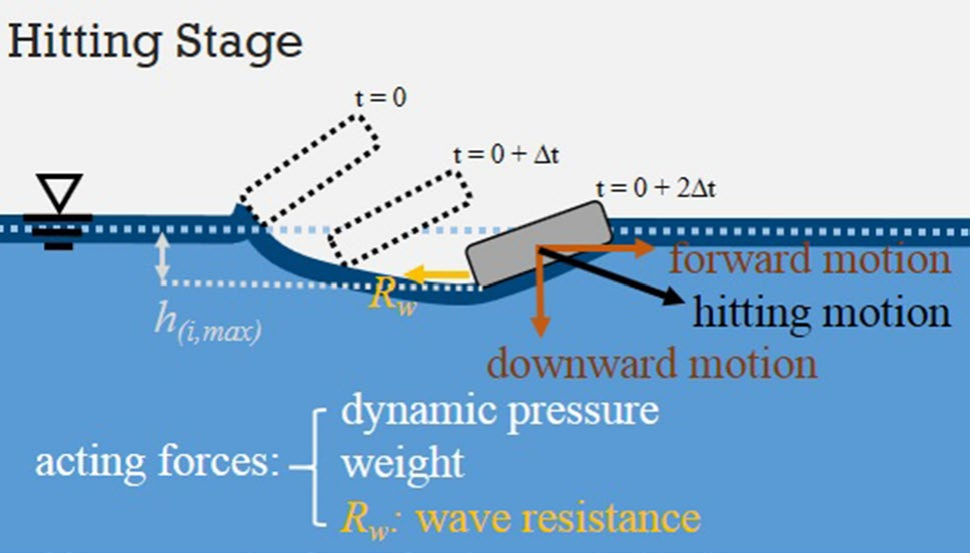
The motion of stone and forces considered in the hitting stage.

In this stage, as that illustrated in Fig. 5, the stone keeps descending right after it hits the water surface and in the meantime moves and pushes the water forward. The sliding motion along the water surface is less important at this stage. Therefore, in this stage, the forces acting on the stone include the weight of the stone itself, the lifting force from the reaction of the water surface, and the capillary-gravity wave drag. The equations of motion are

3 $$m_{s} \frac{{{\text{d}}V_{i,x} }}{{{\text{d}}t}} = - \frac{1}{2}\rho_{w} V_{i,a}^{2} A_{im} \sin \alpha_{i} - R_{w} = - L_{x} - R_{w} ,$$

4 $$m_{s} \frac{{{\text{d}}V_{i,y} }}{{{\text{d}}t}} = - m_{s} g + \frac{1}{2}\rho_{w} V_{i,a}^{2} A_{im} \cos \alpha_{i} = - m_{s} g + L_{y} ,$$

When comparing Eqs. (3) and (4) with Eqs. (1) and (2), one can find the lift and friction coefficients are assumed to be equal to 1 following that of Bocquet^16^, and a capillary-gravity wave resistance (wave drag) term $R_{w}$ is proposed to replace the friction force of $\rho_{w} V_{i,a}^{2} A_{im} \cos \alpha_{i} /2.$ The latter is a novel contribution of this paper and it will be explained in detail afterwards. The two new symbols $L_{x}$ and $L_{y}$ indicate respectively the retardation force and lift force from the dynamic pressure of the water. They will be used for later comparison with other forces by their magnitudes. When solving Eqs. (3) and (4), the initial conditions considered are $\left( {V_{i,x} , V_{i,y} } \right)_{t = 0} = \mathop{V}\limits^{\rightharpoonup} _{i,a}$.

### The sliding stage

**Figure 6:**
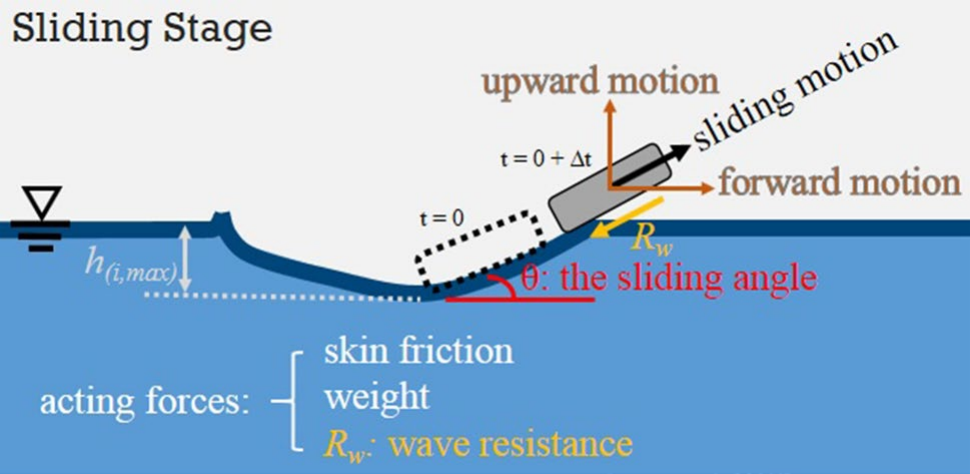
The motion of stone and forces considered in the sliding stage.

In this stage, all forces acting on the stone are illustrated and listed in Fig. 6. The physical mechanism and its interpretation are different from those of the hitting stage. The prominent features in the sliding stage are the followings.

1. The frictional resistance may become important when the stone starts to slide along and finally skip off the water surface.
2. The generated capillary-gravity wave resistance is opposing to the moving (sliding) direction and can be decomposed into (*R*_*w*_)_x_ and (*R*_*w*_)_y_ in *x* and *y* directions when the equations of motion are considered.
3. In this stage, the stone keeps moving forward and in the meantime starts to ascend, i.e., moving away from the water. The reaction force from the water surface does not exist anymore. Instead, the impulse, *I*, on the stone by the reaction force from the water surface during a very small time interval in the hitting stage provides the initial *y*-velocity of the stone in the sliding stage.

With these features, the equations of motion in *x* and *y* directions in the sliding stage are

5 $$m_{s} \frac{{{\text{d}}V_{i,x} }}{{{\text{d}}t}} = \tau A_{im} \cos \theta - \left( {R_{w} } \right)_{x} ,$$

6 $$m_{s} \frac{{{\text{d}}V_{i,y} }}{{{\text{d}}t}} = - m_{s} g + \tau A_{im} \sin \theta - \left( {R_{w} } \right)_{y} .$$

The following compatibility conditions also have to be satisfied:

7 $$V_{i,x} \left( {t = 0} \right)_{{{\text{sliding}}}} = { }V_{i,x} \left( {y = h_{{i,{\text{max}}}} } \right)_{{{\text{hitting}}}} ,$$

8 $$V_{i,y} \left( {t = 0} \right)_{{{\text{sliding}}}} = { }\frac{I}{{m_{s} }}.$$

In the above equations, $\tau$ represents skin frictional stress at the bottom of stone and $\theta$ is the sliding angle when the stone slides along the water surface. It is assumed that the frictional resistance $\tau A_{im}$ prevails over the lift force of Eqs. (3) and (4) and acts in its opposite direction. When comparing Eqs. (6) with (4), one can find the capillary-gravity wave resistance $R_{w}$ appears only in Eq. (6) but not in Eq. (4). The argument is the effect of capillary-gravity wave resistance is less significant in vertical direction during the hitting stage. With regard to the initial conditions of sliding stage in Eq. (7), $h_{{i,{\text{max}}}}$ indicates the maximum descending depth of the stone at the end of hitting stage, which occurs when $V_{{i,{\text{y}}}} \left( {h_{{i,{\text{max}}}} } \right) = 0$ at $t_{{{\text{max}}}}$ after hitting; and $I$ in Eq. (8) indicates the impulse originated from the previous hitting stage, which can be evaluated from

9 $$I = \mathop \smallint \limits_{0}^{{t_{\max } }} \left( {\frac{1}{2}\rho_{w} V_{i,a}^{2} A_{im} \cos \alpha_{i} } \right){\text{d}}t.$$

## Scale analysis and simplification

The estimation of the capillary-gravity wave resistance and shear stress in the above equations of motion needs to be addressed carefully before a solution can be obtained.

### *The capillary-gravity wave resistance*, $R_{w}$

**Figure 7:**
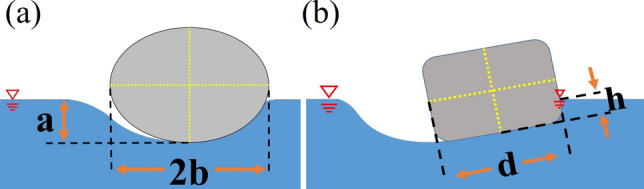
(**a**) The aspect ratio $$\overset{\lower0.5em\hbox{$\smash{\scriptscriptstyle\smile}$}}{\text{A}}$$ is defined as $$a/b$$. (**b**) In the proposed model, $$\overset{\lower0.5em\hbox{$\smash{\scriptscriptstyle\smile}$}}{\text{A}}$$ is defined as $$h/d$$.

The aforementioned capillary-gravity wave resistance on a small object moving along a liquid surface has been studied in detail by Raphael and de Gennes^17^ and later modified by Le Merrer et al.^18^ by adding an aspect ratio to the original mathematical model proposed by Raphael and de Gennes. According to Le Merrer et al. and referring to Fig. 7, one can estimate the capillary-gravity wave resistance exerted on the stone during the hitting and sliding stages as follows.

10 $$R_{w} \sim \left( {\overset{\lower0.5em\hbox{$\smash{\scriptscriptstyle\smile}$}}{\text{A}}} \right)^{2} \frac{{2\rho_{w} m_{s}^{2} g^{2} }}{{3\pi \gamma^{2} }}V_{i,x}^{2} = C\left( {\overset{\lower0.5em\hbox{$\smash{\scriptscriptstyle\smile}$}}{\text{A}}} \right)^{2} \frac{{2\rho_{w} m_{s}^{2} g^{2} }}{{3\pi \gamma^{2} }}V_{i,x}^{2} { ,}$$

where $$\overset{\lower0.5em\hbox{$\smash{\scriptscriptstyle\smile}$}}{\text{A}}$$ is the aspect ratio, $$\gamma$$ is the surface tension of water, and $$C$$ is a proportional constant. The value of $$C$$ depends on the shape of stone and has to be determined experimentally. Following the concept of Le Merrer et al. the aspect ratio $$\overset{\lower0.5em\hbox{$\smash{\scriptscriptstyle\smile}$}}{\text{A}}$$ in this paper is defined in Fig. 7.

### *The skin frictional stress*, $\tau$

The skin frictional stress mentioned in the previous section can be derived by the Kármán-Pohlhausen momentum-integral approach in studying a moving flat plate with velocity $V_{i,x}$. By assuming a quadratic velocity profile of the nearby water, it gives^19^

11 $$\frac{\tau }{{\rho_{w} }} = \frac{1}{5}\frac{{\text{d}}}{{{\text{d}}x}}\left( {V_{i,x}^{2} \delta } \right),$$

where $\delta$ is the boundary thickness. Since the dimensional size of the stone is usually small, the variation of $\delta$ is negligible and hence

12 $$\delta = \delta_{ave} \approx \frac{d}{{\sqrt {R_{e} } }} = \frac{d}{{\sqrt {V_{i,x} \left( {h_{\max } } \right)\left( {\frac{d}{\nu }} \right)} }},$$

in which $d$ is the characteristic dimension of the stone as that shown in Fig. 7, ${R}_{e}$ is the Reynolds number, and $\nu$ is the kinematic viscosity of water. It then yields

13 $$\tau \approx \frac{{\rho_{w} \delta_{ave} }}{5}\frac{{\text{d}}}{{{\text{d}}x}}\left( {V_{i,x}^{2} } \right).$$

In order to simplify the equations of motion of the previous section furthermore, the relative magnitudes the capillary-gravity wave resistance $R_{w}$, the retardation force $L_{x}$, and the frictional resistance $\tau A_{im}$ are discussed in detail through scale analysis. The result can be used for determining the relative importance of those terms and simplifying our numerical algorithm for solutions. The scale analysis is carried out based on experimental data obtained by Tsai and his associates^19,20^ where the stone is resembled by a circular disk. Let the diameter of disk $d$ and the initial throwing velocity $V_{0}$ be characteristic length and characteristic velocity of the stone respectively, it is found from our experiment that

14 $$\frac{{R_{w} }}{{L_{x} }}{ }\sim { }10^{2} ,$$

And

15 $$\frac{{R_{w} }}{{\left| \tau \right|A_{0} }}{ }\sim { }10^{3} ,$$

where $A_{0} = \pi d^{2} /4$ is the characteristic area of the circular disk resembling the stone.

From the above scale analysis, it can be learned that among the three forces $R_{w}$, $L_{x}$ and $\left| \tau \right|A_{0}$, the capillary-gravity wave resistance is the dominant one. Therefore, the equations of motion of the stone in the hitting stage can be simplified from Eqs. (3) and (4) to be

16 $$m_{s} \frac{{{\text{d}}V_{i,x} }}{{{\text{d}}t}} \approx - R_{w} ,$$

17 $$m_{s} \frac{{{\text{d}}V_{i,y} }}{{{\text{d}}t}} \approx - m_{s} g + L_{y} ,$$

in the hitting stage, and

18 $$m_{s} \frac{{{\text{d}}V_{i,x} }}{{{\text{d}}t}} \approx - (R_{w} )\cos \theta ,$$

19 $$m_{s} \frac{{{\text{d}}V_{i,y} }}{{{\text{d}}t}} \approx - m_{s} g - (R_{w} )\sin \theta ,$$

in the sliding stage. The above two sets of equations are quite different from the equations of motion proposed by Bocquet^16^ where the capillary-gravity wave resistance is neglected completely and the same set of equations is applied throughout the entire skipping process.

In “Numerical algorithm” Section, the mathematical formulation derived above will be applied to depict mathematically the experiments of stone-skipping process carried out by Tsai^19^ to justify its appropriateness.

## Numerical algorithm

**Figure 8:**
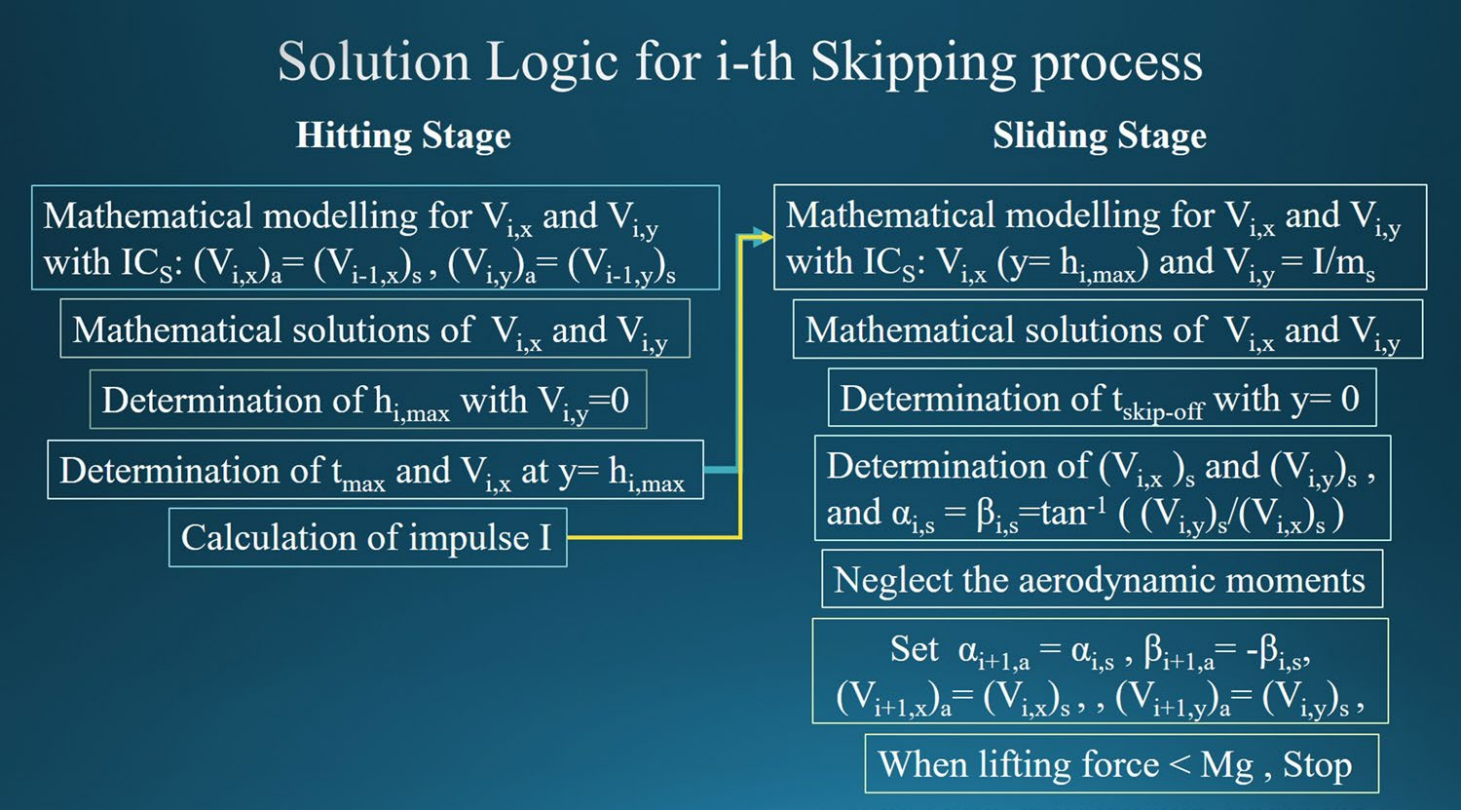
Numerical algorithm containing consecutive skips in the stone-skipping process.

By neglecting aerodynamic and a few hydrodynamic forces and moments exerted on the stone, the mathematical formulation derived in the previous two sections allows us to describe the stone-skipping process and predict its results in advance. In particular, given a specified initial throwing condition, the number of skips can be predicted through the algorithm of Fig. 8 which is established based on the mathematical formulation. It should be mentioned that subscript a in Fig. 8 is used to indicate physical quantities when the stone is approaching the water and s indicates quantities when the stone is skipping-off (leaving) the water. The algorithm of Fig. 8 can be used for describing the 1st skip of a stone arisen from the initial throw of a person as well as the consecutive skips. Iterative calculations are used extensively in the algorithm. The computational process ceases when the lift force is smaller than the weight of stone indicating the end of the stone-skipping process. The total number of skips can be predicted accordingly. Owing to the page limit, Fig. 8 may not offer enough information that relates the algorithm to the equations listed above. Interested readers are suggested to refer to Tsai^19^ for its details.

In the next section, the proposed mathematical formulation and numerical algorithm will be tested by the experimental results of Tsai and his associates^19,20^.

## Verification by experimental result

**Figure 9:**
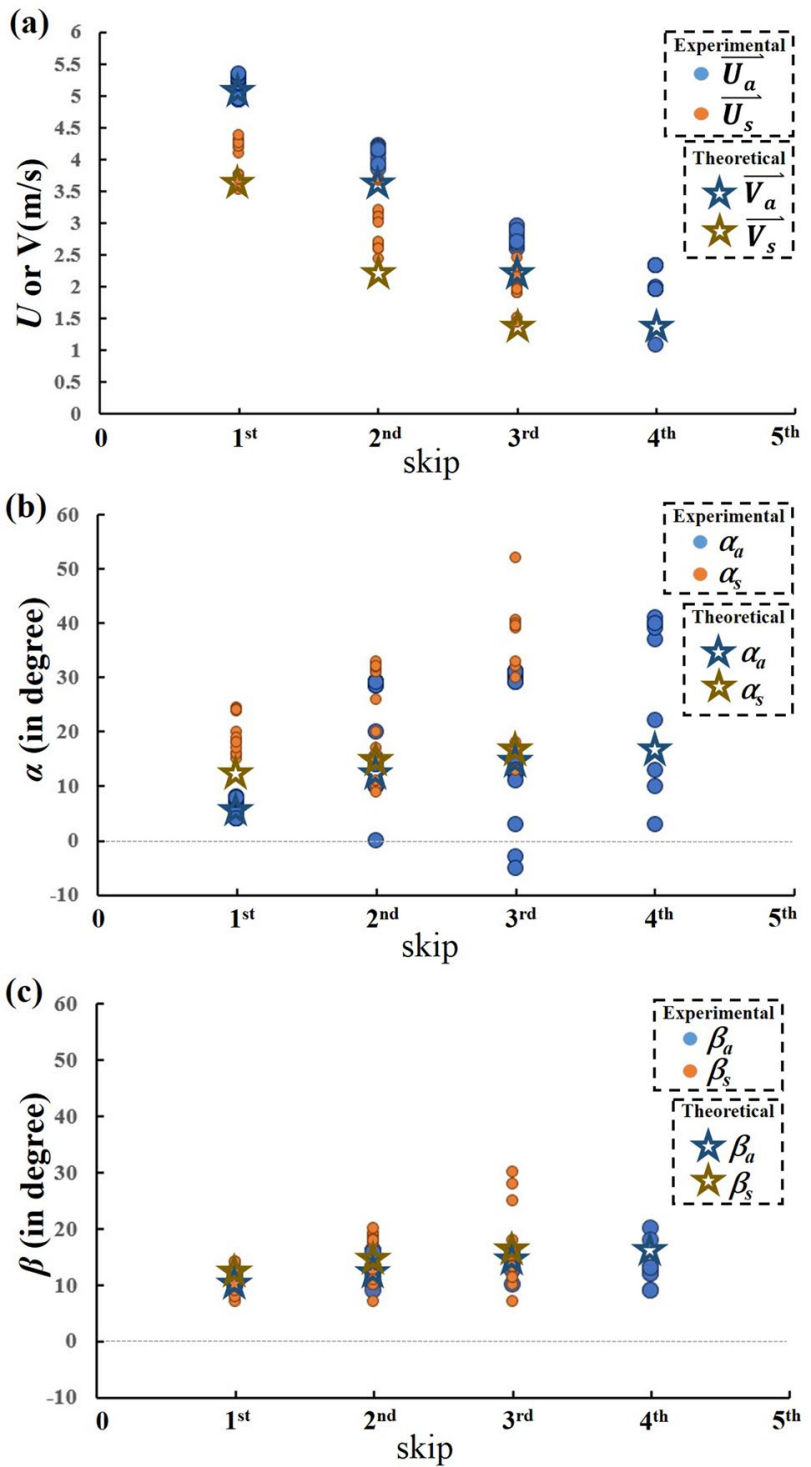
Comparison of numerical results ( and ) with experimental data ( and ): (**a**) velocity, (**b**) incline angle, and (**c**) moving angle of the stone at each skip.

The parametric values employed in this study are shown in Table 1. For initial conditions, the inclined angle of throw was kept within the range of 4° to 6° and the approaching velocity $V_{0}$ was kept to be around 5 m/s in our experimental work. However, the initial approaching angle (*β* in Fig. 1 but with a subscript 0) was kept to be 10°. A few results obtained following the numerical algorithm of Fig. 8 are shown in Fig. 9 and marked with asterisks. Their corresponding data measured from 78 experiments^19,20^ are also plotted in the figure and denoted by dots. In particular, the velocity $V$ (or *U* for experimental result), inclined angle $\alpha$, and moving angle $\beta$ of the stone at each skip are shown in Fig. 9a,b and c, respectively. For the latter two angles, subscripts “*a*” and “s” are used to describe when the stone is “approaching” or “skipping off” the water surface. Since it was not easy to control the initial throwing conditions and measure the angle and velocity precisely in experiments, and also because there are many data points, the dots scatter to a certain extent at each skip in the figure. However, there are only two data points for their corresponding numerical results indicating, respectively, the approaching and skipping off of the stone. In can be seen from the figures the stone sinks into water after 4 skips. It is also found the agreement between the numerical results and the experimental data is surprisingly good.

**Table 1:** Parametric values in this study.

$m_{s}$	$d$	$\gamma$	$\nu$	$\rho_{W}$	$g$
$0.009\;{\text{kg}}$	$0.03\;{\text{m}}$	$7.34 \times 10^{ - 2} \;{\text{kg}}/{\text{s}}^{2}$	$1.12 \times 10^{ - 3} \;{\text{kg}}/{\text{ms}}$	$999\;{\text{kg}}/{\text{m}}^{3}$	$9.8\;{\text{m}}/{\text{s}}^{2}$

After the above justification, it is argued the proposed numerical algorithm can be used to predict the number of skips with a specific initial condition. For example, under initial conditions of $\alpha_{0} = 5^\circ { },\beta_{0} = 10^\circ { },\;{\text{and}}\;V_{0} = 5, \;10, \;15,\; {\text{and }}\;20\; {\text{m}}/{\text{s}}$ respectively, the numbers of skips are 4, 5, 6, and 7 respectively. Based on our experimental and numerical experience, the number of skips is affected predominantly by the throwing velocity but not $\alpha_{0}$ and $\beta_{0}$.

## Concluding remarks

The non-spinning stone-skipping process has been studied with a viewpoint different from what have been known before. Specific features of the present study are as follows.

1. Although it can be divided into several stages when a stone hits and then leaves the water surface, the major part consists of a hitting stage and a sliding stage. Since forces acting on the stone are not quite the same in these two stages, mathematical models are formulated differently in these two stages in this paper, and it is the major different from other studies.
2. The capillary-gravity wave resistance is included in the formulation for the first time. It is found to be the dominant factor based on result of the scale analysis of this paper.
3. The above scale analysis also reveals the relative importance of various forces acting on the stone in the mentioned two stages. Mathematical formulation and numerical algorithm can be simplified accordingly.
4. A numerical algorithm is proposed to describe the entire stone-skipping processes from the first hitting of water to the sink of stone. The algorithm includes parameters of the throwing velocity, inclined angle and moving angle. And the total number of skips can be predicted accordingly once an initial throwing condition is specified.
5. The moderately good agreement between the prediction result and experimental measured data indicates the applicability of the proposed mathematical formulation and numerical algorithm.

To further improve the formulation, spinning moments exerted on the stone during both the hitting and sliding stages, and the aerodynamic forces and moments acting on the stone while it flies through the air can be taken into consideration. It would make the stone-skipping modeling more accurately at the expense of mathematical tediousness.

## Data Availability

The datasets used and/or analyzed during the current study are available from the corresponding author on reasonable request.
